# Supplementary material for: Evidence supported by Mendelian randomization: impact on inflammatory factors in knee osteoarthritis
Source: Front Med (Lausanne). 2024 May 28;11:1382836. doi: 10.3389/fmed.2024.1382836 (PMC11165061; doi:10.3389/fmed.2024.1382836)
Supplement: Supplementary file 1 [file Data_Sheet_1.ZIP › data/GCST90274847_5e6/847.leaveoneout.pdf]

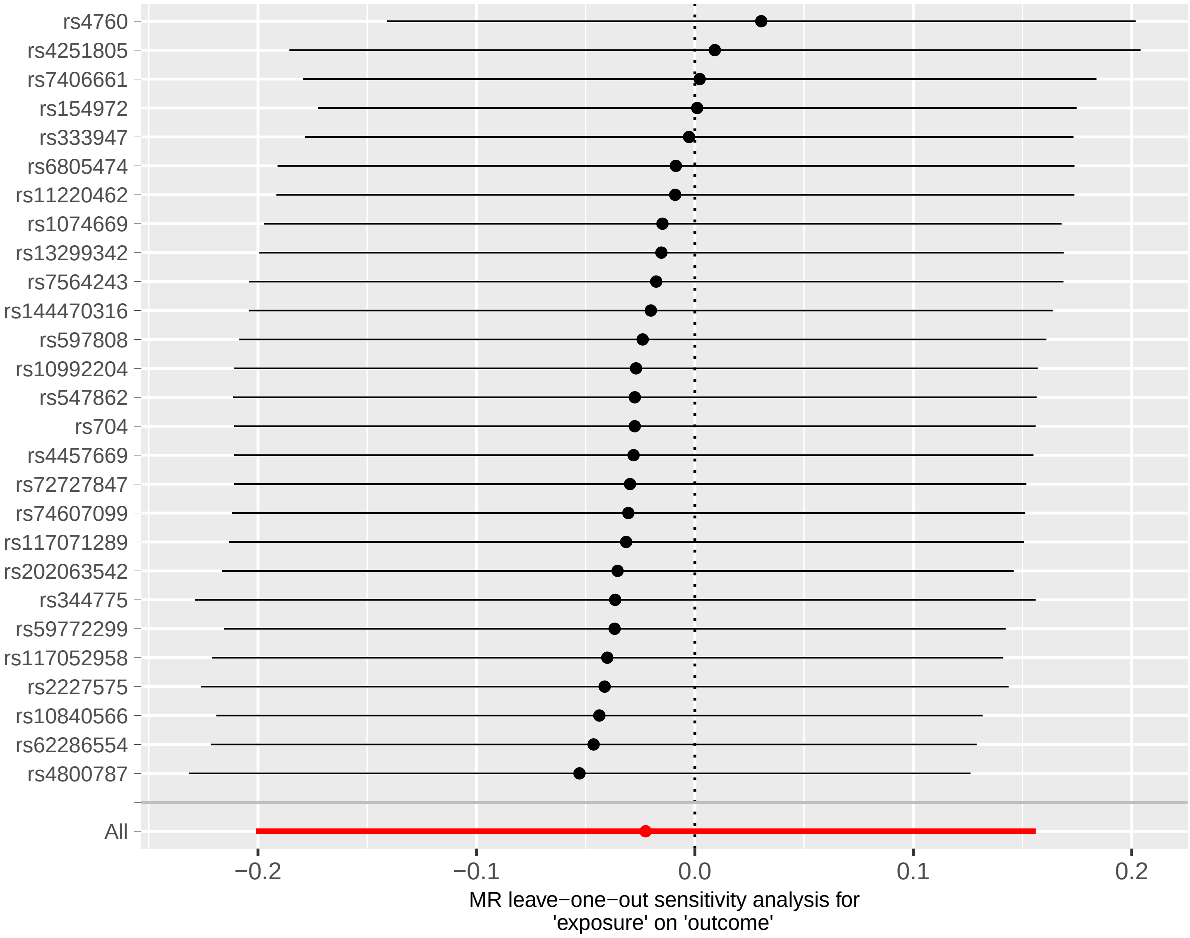

rs4760
rs4251805
rs7406661
rs154972
rs333947
rs6805474
rs11220462
rs1074669
rs13299342
rs7564243
rs144470316
rs597808
rs10992204
rs547862
rs704
rs4457669
rs72727847
rs74607099
rs117071289
rs202063542
rs344775
rs59772299
rs117052958
rs2227575
rs10840566
rs62286554
rs4800787
All
−0.2
−0.1
0.0
0.1
0.2
MR leave−one−out sensitivity analysis for
'exposure' on 'outcome'
